# Impact of fetal echocardiography

**DOI:** 10.4103/0974-2069.52806

**Published:** 2009

**Authors:** John M Simpson

**Affiliations:** Director of Pediatric Echocardiography, Department of Congenital Heart Disease, Evelina Children's Hospital, Guy's and St Thomas' NHS Foundation Trust, London, UK

**Keywords:** Congenital heart disease, echocardiography, fetal heart

## Abstract

Prenatal diagnosis of congenital heart disease is now well established for a wide range of cardiac anomalies. Diagnosis of congenital heart disease during fetal life not only identifies the cardiac lesion but may also lead to detection of associated abnormalities. This information allows a detailed discussion of the prognosis with parents. For continuing pregnancies, appropriate preparation can be made to optimize the postnatal outcome. Reduced morbidity and mortality, following antenatal diagnosis, has been reported for coarctation of the aorta, hypoplastic left heart syndrome, and transposition of the great arteries. With regard to screening policy, most affected fetuses are in the “low risk” population, emphasizing the importance of appropriate training for those who undertake such obstetric anomaly scans. As a minimum, the four chamber view of the fetal heart should be incorporated into midtrimester anomaly scans, and where feasible, views of the outflow tracts should also be included, to increase the diagnostic yield. Newer screening techniques, such as measurement of nuchal translucency, may contribute to identification of fetuses at high risk for congenital heart disease and prompt referral for detailed cardiac assessment.

## THE POTENTIAL FOR DETECTION OF CONGENITAL HEART LESIONS BY FETAL ECHOCARDIOGRAPHY

A wide range of congenital heart defects can be identified during fetal life with a very high degree of diagnostic accuracy in specialist centers.[1] Virtually all forms of congenital heart disease have been described during prenatal life. In most countries, the major routes of ascertainment of congenital heart defects are, first, a suspicion of a cardiac defect during an obstetric anomaly scan, or second, because specific risk factors have led to a referral to a specialist unit for further evaluation. The types of risk factors that are widely accepted as referral reasons for detailed assessment of the heart are listed in [Table 1] and have been reviewed elsewhere[2] It should be emphasized that most cases of congenital heart disease occur in the “low risk” population. Detection of these cases rests with the sonographer who is assessing the fetal heart as part of obstetric anomaly scans involving an anatomic survey that is not restricted to the heart. Some cardiac lesions, particularly those evident on a “four chamber view” of the fetal heart are more easily detected by the nonspecialist sonographer than others, for which more extended views of the outflow tracts are required for detection. For example, United Kingdom national data obtained between 1993 and 1995 reported a detection rate of 38% for atrioventricular septal defects and 66% for hypoplastic left heart (“four chamber” abnormalities) compared to 3% for transposition of the great arteries (outflow tract views required for detection).[2,3] [Table 2] summarizes lesions which should be evident on four chamber screening, those which require views of the outflow tracts, for recognition, lesions which are considered very difficult to detect even in specialist hands, and those which cannot be detected during fetal life.

**Table 1:** Summary of risk factors that should prompt detailed cardiac evaluation

Fetal factors
Suspected cardiac abnormality on screening ultrasound
Increased nuchal translucency thickness
Fetal hydrops
Fetal abnormality with known association of congenital heart disease
e.g., exomphalos, diaphragmatic hernia
Fetal arrhythmia
Abnormal fetal karyotype, e.g., trisomy 21
Maternal and familial risk factors
Family history of congenital heart disease (CHD) in a first degree relative.
Diabetes mellitus – Mothers who are established diabetics on treatment
Mothers with a tendency toward diabetes that is solely related to pregnancy are not judged candidates for fetal echocardiography
Mothers taking known teratogenic drugs, e.g., anticonvulsants, lithium
Maternal anti - Ro or anti - La antibodies
Mothers who have anti-Ro and / or La antibodies are candidates for fetal cardiology assessment, in view of the risk of developing fetal heart block. Mothers NOT having anti Ro or La antibodies are not candidates for fetal echocardiography
Maternal infections, e.g., parvovirus, Coxsackie

**Table 2:** Summary of the scope and limitations of fetal echo in the diagnosis of commonly occuring major cardiac malformations

Examples of major lesions evident on “Four chamber views” of the fetal heart
Hypoplastic left heart syndrome
Severe coarctation of the aorta
Critical aortic stenosis
Tricuspid atresia
Pulmonary atresia with intact ventricular septum
Atrioventricular septal defect
Double inlet ventricles
Examples of major lesions where the four chamber view of the heart is typically normal / near normal and for which views of the outflow tracts are required
Transposition of the great arteries
Tetralogy of Fallot + / - pulmonary atresia
Common arterial trunk
Some forms of coarctation of the aorta
Examples of lesions that are difficult to detect even in experienced hands
Total anomalous pulmonary venous drainage
Coarctation of the aorta (milder forms)
Some types of ventricular septal defect
Milder forms of aortic and pulmonary valve stenosis
Lesions that cannot be predicted from prenatal cardiac imaging
Patent arterial duct
Secundum atrial septal defects

## IMPACT OF FETAL ECHOCARDIOGRAPHY ON PREVALENCE OF CONGENITAL HEART DISEASE

When congenital heart disease is diagnosed during fetal life, the expectant parents should have a detailed discussion with a fetal cardiologist with regard to the prognosis of the cardiac lesion, covering not only procedural risks, but also long-term mortality, morbidity, and quality of life. There should also be a discussion with regard to possible associations, including karyotypic abnormalities, noncardiac structural anomalies, and syndromes,[2–5] to have a full picture of the prognosis for their baby. Thus, effective management demands a close liaison between the cardiologist, fetal medicine specialist, genetics, and other relevant subspecialities. Depending on the severity of the cardiac lesion, the associated abnormalities, gestational age, and local laws, one of the options open to parents may include termination of pregnancy. It is self-evident that if congenital heart disease is diagnosed prenatally and parents elect to terminate the pregnancy, then the prevalence at birth will fall. The proportion of parents who elect to terminate the pregnancy will depend on many factors including religion and cultural norms, as well as, the prognosis of the cardiac lesion and any associated abnormalities. As examples, if hypoplastic left heart is diagnosed then, at my center, over 60% of the parents will elect to terminate the pregnancy, whereas, only a small minority of parents would consider this option for isolated transposition of the great arteries. It should be emphasized that the decision about the fate of the pregnancy rests with the parents after discussion with their medical advisers, and it is not our practice to direct parents as to whether they should or should not continue with pregnancy. Parental decision-making may, however, be constrained by local laws. For example, in the United Kingdom, termination of pregnancy may, among other reasons, be performed if “there is a substantial risk that if the child were born it would suffer from such physical or mental abnormalities as to be seriously handicapped.” Reliable population-based data regarding the impact of prenatal diagnosis on birth prevalence is relatively scant. In the United Kingdom, between 1993 and 1995 such national data was collected.[2,3] Overall, around half of the pregnancies affected by fetal congenital heart disease ended in termination of pregnancy, although the overall detection rate of congenital heart defects (requiring intervention or surgery in infancy) was only 24%. Thus, if around one-quarter of the affected pregnancies were detected and half of these resulted in termination of pregnancy, then around one-eighth fewer infants might be delivered. Other notable findings from that UK national data include the wide geographical variation in detection rates and that the termination rate was affected by the gestational age at which the diagnosis was made. Early detection was associated with a higher termination rate than diagnoses made later in gestation. More recent European data has confirmed major differences in detection rates between different European countries and emphasized the importance of noncardiac malformations and karyotypic abnormalities in prenatal detection rates and parental decision-making.[6–8]

Since those studies were published there have been dramatic changes in screening policy for congenital heart disease. Although established risk factors for congenital heart disease, such as a history in a first-degree family member, have been recognized for some time, nuchal translucency (NT) screening is a more recent development, which has far-reaching implications for cardiac screening policies. Nuchal translucency screening involves measurement of a sonographically lucent area at the back of the fetal neck. This technique was introduced to identify fetuses at high risk for trisomy 21, but NT thickness has a correlation with congenital heart disease (CHD), which is independent of the fetal karyotype[9] and has a stronger association with CHD than established risk factors such as family history.[10] From the data of Hyett *et al*.,[9] 6.3% of fetuses with NT above the ninety-ninth percentile (3.5 mm) had congenital heart disease. This is not a simple “cut-off” relationship, but correlates with the NT measurement, the higher the NT thickness, the higher the risk of CHD.[11] The NT measurements are made at 11-14 weeks gestational age and so fetal echocardiography may be indicated shortly thereafter. Some data does not suggest as strong a relationship of NT to congenital heart disease as the original data of Hyett *et al*.[9,12,13] A recent multicenter study suggested that around one-quarter of chromosomally normal fetuses with congenital heart disease have an NT value above 3.5mm.[14] This has led to early identification of CHD[14] and an increased demand for early fetal echocardiography on the basis of such findings.[15,16] The policy at my unit has been to await fetal karyotype results before performing fetal echocardiography because some parents may base their decision regarding the fate of the pregnancy on the karyotype result alone rather than on the cardiac findings. Other fetal cardiologists, practicing within fetal medicine units are examining the heart even earlier at around 11-13 weeks gestational age, targeting fetuses with increased NT.[17]

## IMPACT OF PRENATAL DIAGNOSIS ON CARDIAC MORBIDITY AND MORTALITY

The vast majority of data on the impact of prenatal diagnosis on the morbidity and mortality of congenital heart disease is from developed countries. The data published to date will be reviewed briefly, but it should be emphasized that all the data comes from countries where the following requirements were met:

- Availability of appropriate prenatal investigations: If a cardiac lesion was diagnosed prenatally there was availability of relevant prenatal investigations, such as, fetal karyotyping and detailed ultrasound for noncardiac malformations.
- High level delivery facilities and neonatal care was available: Following a prenatal diagnosis of congenital heart disease there would need to be a pattern of referral for delivery and treatment at a high level neonatal nursery/cardiac center if the outcome was to be optimized.
- Postnatal surgical, interventional or medical treatment for the cardiac lesion in question was available without this being financially prohibitive.
- Availability of long-term therapies: Prenatal diagnosis has an ascertainment bias for more severe forms of congenital heart disease. Cardiac lesions which will lead to single ventricle palliation are overrepresented in prenatal versus postnatal series. Any country or region instituting a prenatal screening program will have to consider which surgical options are available, for example, total cavopulmonary connection, both in terms of surgical feasibility and supportive care, for example, monitoring of anticoagulation.
- Program for postnatal detection of congenital heart disease: If a diagnosis of congenital heart disease is not made prenatally, in developed countries there is typically a program of clinical assessment for newborn infants, which includes the cardiovascular system, accepting that some lesions will be extremely difficult to detect clinically in the first days after birth. In some developing countries, early postnatal assessment may not be performed routinely and so CHD may remain undetected for a longer period, and duct-dependent lesions may not be detected prior to death. In this context, prenatal diagnosis may have a more dramatic impact in developing countries than in developed countries.

The lesions for which there is relatively robust published data relates to hypoplastic left heart syndrome, transposition of the great arteries, coarctation of the aorta, and pulmonary atresia.

### Hypoplastic left heart syndrome

Tworetzky *et al*. (2001)[18] described a series of 33 fetuses who were diagnosed prenatally with hypoplastic left heart and who were managed at a single center in the United States. They described zero mortality among 14 prenatally diagnosed infants versus a mortality of 13 of 38 infants who were diagnosed postnatally. In addition to mortality benefit, prenatal diagnosis of hypoplastic left heart was associated with better ventricular function, less tricuspid regurgitation, and a reduced requirement for inotropes and bicarbonate. Other data has confirmed better condition at presentation for infants who were diagnosed prenatally, but this did not lead to reduced overall mortality.[19] A further publication has reported a reduced incidence of abnormal neurological events related to prenatal diagnosis.[20]

### Transposition of the great arteries

The largest series examining the impact of prenatal diagnosis of transposition of the great arteries (TGA) on the outcome is from France, where there is a very well-developed prenatal screening program for congenital heart disease.[21] The study included 68 infants who were prenatally diagnosed and 250 who were not. There was zero preoperative mortality in the prenatal group versus 6% in the postnatally diagnosed group. Postoperative mortality was also significantly better for infants who were diagnosed during fetal life. Another study, however, did not observe such an impact of prenatal diagnosis on the condition at presentation or on the operative mortality.[19]

### Coarctation of the aorta

A single study has examined the impact of prenatal diagnosis of coarctation of the aorta on the postnatal outcome.[22] This study reported a positive impact of prenatal diagnosis in terms of preoperative morbidity and mortality as well as ventricular function at presentation. Importantly, this study included pathological data relating to infants who died prior to diagnosis. This type of information is difficult to obtain unless there is a population-based pathological registry. Where such pathological data has been reported, there is an important minority of infants who die prior to diagnosis,[23] even in the setting of a developed country.

### Pulmonary atresia

My center has published data on the impact of prenatal diagnosis on the outcome of infants with duct-dependent pulmonary blood flow.[24] The prenatally diagnosed infants had better oxygen saturation at presentation than those diagnosed postnatally. However, this did not translate into better short-term mortality or morbidity. In our series of pulmonary atresia, almost all infants were detected postnatally in the first 24 hours after birth, with prompt initiation of prostaglandin E. If there had not been such prompt recognition of cases that were undiagnosed prior to birth, then the results may well have been more favorable toward a benefit of prenatal diagnosis. Thus, the results are likely to be strongly influenced by postnatal screening policies as well as prenatal detection.

## IMPACT OF PRENATAL DIAGNOSIS FOR MANAGEMENT STRATEGIES

There are many cardiac lesions for which prenatal diagnosis has little impact on initial neonatal management. Examples include isolated ventricular septal defect and atrioventricular septal defect, that is, those cases without evidence of left or right heart obstruction, which would not be expected to present until the pulmonary vascular resistance falls postnatally. For these cases, there is no cardiac indication to alter delivery plans, provided the need for non-urgent cardiac assessment in the neonatal period is understood.

### Location of delivery

For fetuses who have duct-dependent lesions or where there is a potential need for early neonatal surgery or intervention, delivery at or near a cardiac center may be preferable so that prompt postnatal investigations can be planned without the need for transfer of the infant. Importantly, this also ensures that parents are available for explanation and consent for early neonatal procedures. Ensuring delivery at the cardiac center may involve induction of labor at term, but the vast majority of infants can have a vaginal delivery rather than a Caesarean section. In practice, however, many parents may favor delivery at or near a cardiac center, even if the cardiac findings are not suggestive of the need for very early intervention. This largely reflects local facilities and parental concerns about separation from their newborn infant if assessment at a geographically remote cardiac center (even if non-urgent) has been recommended.

## IMPACT ON MODE OF DELIVERY

For most cardiac lesions, given prenatal circulatory physiology, a normal vaginal delivery is perfectly satisfactory. In this author's practice Caesarean delivery has been reserved for a minority of cases, in whom there is a predicted need for early neonatal intervention, where it is necessary to have a team immediately available for the resuscitation and immediate cardiac management of the affected fetus. This has included infants with TGA with both a restrictive atrial septum and restrictive arterial duct, fetuses with hypoplastic left heart with restrictive/intact atrial septum, and hydropic fetuses where immediate fluid drainage from body cavities such as the pleural space may be indicated urgently. In such cases immediate cardiological, interventional, and/or cardiac surgical availability needs to be ensured. There should be an individualized discussion between obstetrician, cardiologist, and cardiac surgeon, to optimize care. Aside from fetuses with structural cardiac malformations, some types of arrhythmia such as complete heart block may make it impossible to assess fetal well-being by conventional cardiotocographic monitoring and Caesarean section may be preferred.

### Prenatal intervention

Prenatal diagnosis affords a unique opportunity to intervene and alter the natural history of cardiac disease. In this context, it is essential to separate abnormalities of cardiac rhythm and structural abnormalities.

- *Fetal arrhythmias*: For fetal tachycardias, which are most commonly supraventricular tachycardia or atrial flutter, there is ample evidence of the effectiveness of prenatal therapy to control the arrhythmia and lead to the resolution of fetal hydrops if present.[25–29] The type of therapy used needs to be tailored according to the type of arrhythmia and the presence of hydrops (which affects the placental transfer of drugs). With regard to fetal bradycardia, due to a complete heart block, treatments are more controversial. If the cardiac structure is abnormal, the most common associated abnormalities include isomerism of the left atrial appendages and discordant atrioventricular connections. The prognosis for such fetuses, affected by both structural cardiac disease and complete heart block is guarded, with a minority of fetuses surviving.[30–33] Heart block with a normal cardiac structure is due to maternal anti-Ro or anti-La antibodies in the vast majority of cases. Prenatal therapy for such cases is controversial with some groups recommending therapy such as dexamethasone and salbutamol for all cases, and others treating only the affected fetuses where there is evidence of hemodynamic compromise.[31,34–37]
- *Structural abnormalities*: Prenatal intervention for structural cardiac malformation remains controversial. The lesions for which intervention has been undertaken during fetal life include, critical aortic stenosis,[38–40] pulmonary atresia,[41] and hypoplastic left heart with intact atrial septum.[42] Using critical aortic stenosis as an example, the rationale for intervening by intrauterine balloon aortic valvuloplasty has been to prevent deterioration in the left heart structures, which is recognized to be a part of the natural history in utero.[43,44] Despite technical success, and improvements in the echocardiographic parameters the data on clinical outcome has been less convincing, with only a minority of cases achieving a biventricular repair.[40,45] Currently, such interventions are undertaken at specialist centers, where there is the availability of fetal medicine, fetal cardiology, interventional, surgical, and anesthetic expertise, which are essential for procedural success, follow-up, and postnatal management of the affected infants.

## HOW AND HOW FAR TO TRAIN SONOGRAPHERS TO EXAMINE THE FETAL HEART

From an epidemiological perspective, although groups at high risk for congenital heart disease may be identified, for example, increased NT or family history, most congenital heart lesions will occur in the “low-risk” population. Prenatal detection of CHD in the low-risk population will be dependent on the ability of sonographers to identify deviation from normality on midtrimester anomaly scans. Given the incidence of individual cardiac lesions, sonographers practising in a low-risk setting are unlikely to become familiar with a broad spectrum of congenital cardiac malformations. The goal of training is to ensure that sonographers are familiar with normal cardiac appearances and refer cases which do not fit into the normal pattern. Such referrals are made to specialists who can then provide a precise diagnosis, prognosis, and formulate a management plan for the affected fetus.

One of the key views of the fetal heart is the “four chamber view,” which underpins effective prenatal cardiac screening. This view has the advantage of having external reference points, the fetal ribs, to ensure that the sonographer has “cut” the thorax in the appropriate plane. In a correct four chamber view there should be the appearance of a single rib around the fetal thorax [Figure 1b]. As a minimum, a four chamber view should be obtained, when the heart is imaged as part of “routine” obstetric anomaly scanning in the midtrimester. From the published data, the yield of congenital heart defects increases if the outflow tracts are examined, as well as the four chamber view, but appreciation of abnormalities of the outflow tracts is more challenging than the four chamber view. The approach that we have adopted, in common with others,[46–49] has been to advocate visualization of five key sonographic views [Figure 1a–e]. All these views can be obtained by cranial or caudal angulation of the ultrasound probe from the four chamber view. Examples of cardiac abnormalities that can be suspected on the four chamber view are shown in [Figure 2a–c]. Some cardiac lesions that are compatible with a normal four chamber view and for which extended views of the outflow tracts are required to make the diagnosis are illustrated in [Figure 3a–c]. For fetuses with major congenital heart disease, a full diagnosis requires a sequential segmental approach,[50] similar to postnatal practice.

**Figure 1(b):**
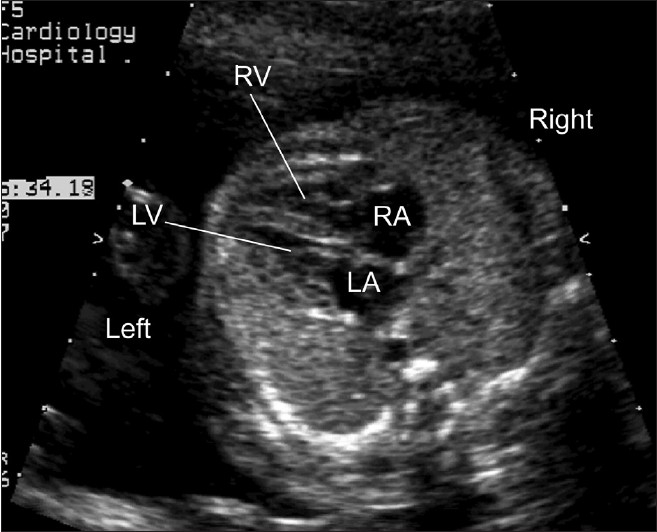
Normal four chamber view. The apex of the fetal heart is anterior and to the left. The heart occupies around one-third of the area of the fetal thorax. A single rib is seen around the fetal thorax confirming that this is a properly orientated four chamber view. The left ventricle (LV) and right ventricle (RV) are of similar diameter. The left atrium (LA) is the closest cardiac chamber to the fetal spine. The right atrium (RA) is of similar size to the left atrium

**Figure 1(a):**
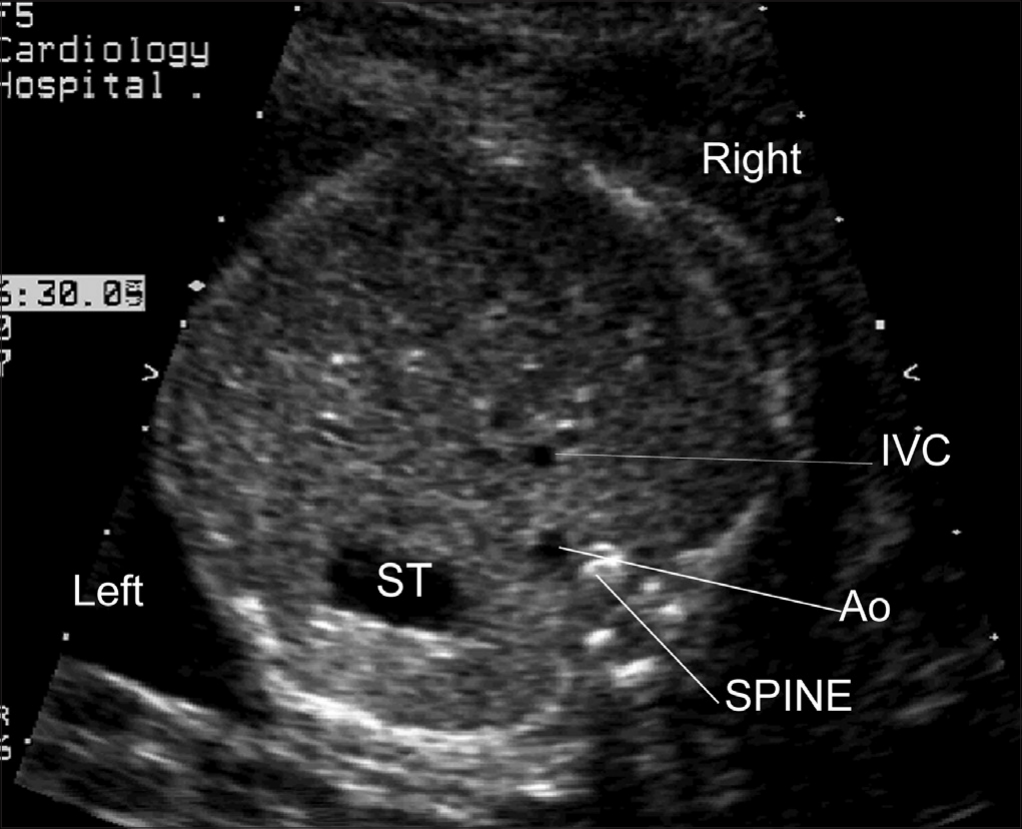
Normal cardiac situs; The fetal stomach (ST) is seen on the left. The descending aorta (Ao) is anterior and to the left of the fetal spine. The inferior vena cava is anterior and to the right of the aorta. (These figures are sequential views commencing with views of the cardiac situs (inferior) and ending with the “three vessel view” in the superior mediastinum)

**Figure 1(c):**
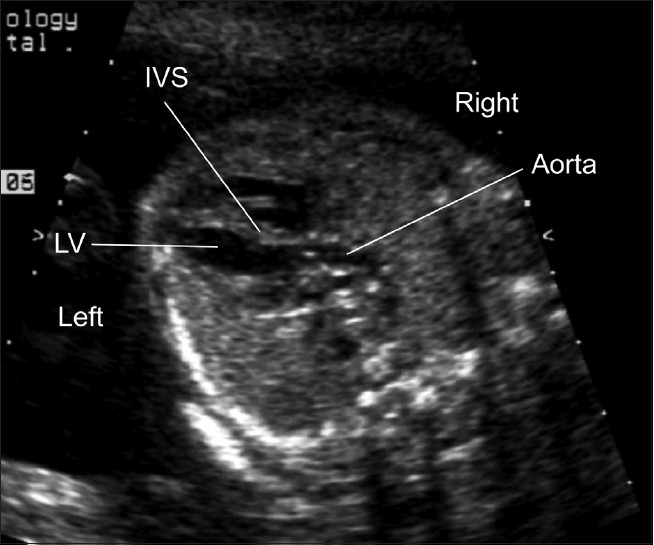
Normal left ventricular outflow tract. The aorta arises from the left ventricle (LV) and heads towards the right shoulder of the fetus. Note that the interventricular septum (IVS) is continuous with the anterior wall of the aorta

**Figure 1(d):**
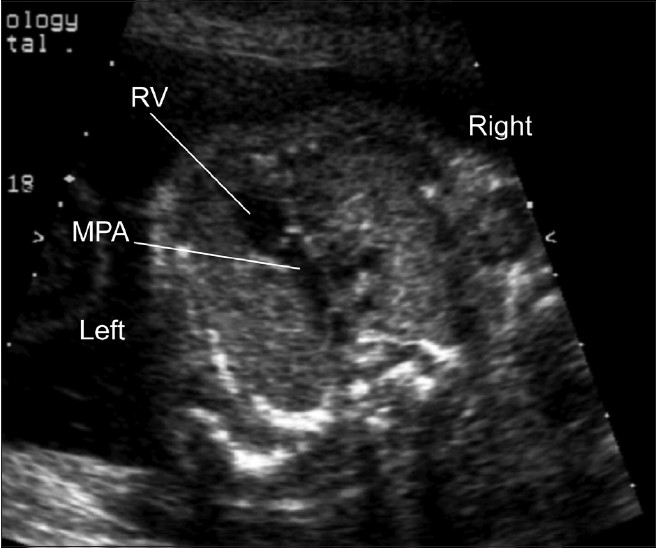
Normal pulmonary artery. The main pulmonary artery (MPA) arises anteriorly from the right ventricle (RV) and passes directly posterior toward the fetal spine. Thus, its orientation is completely different from the aorta as it leaves the heart. This “crossing” pattern of the normally related great arteries is an important feature for the sonographer to note during examination of the outflow tracts

**Figure 1(e):**
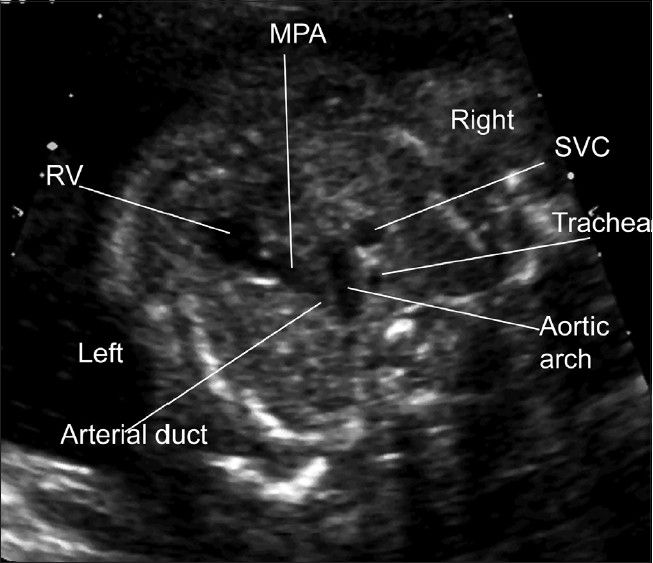
Normal “Three vessel view”. The “three vessel view” refers to a view of the great vessels in the superior mediastinum. The main pulmonary artery (MPA) may be seen passing directly posterior where it meets the arterial duct, which connects to the descending aorta. The transverse aortic arch meets the duct to form a V shape. To the right of the aortic arch there is the circular cross-section of the superior vena cava. Note that the trachea is seen in this projection and normally lies outside the V formed by the transverse aortic arch and the arterial duct

**Figure 2(a):**
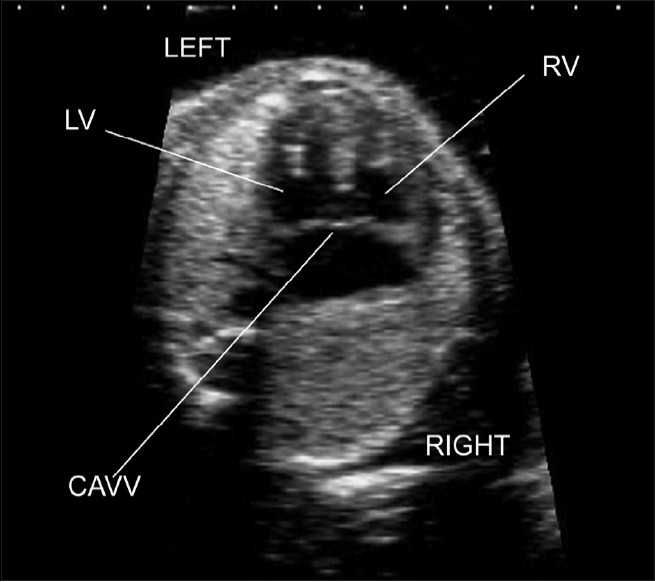
Complete atrioventricular septal defect. This defect is evident on a four chamber view. There is a common atrioventricular junction and in this example there is a large ventricular component and atrial component to the defect

**Figure 2(b):**
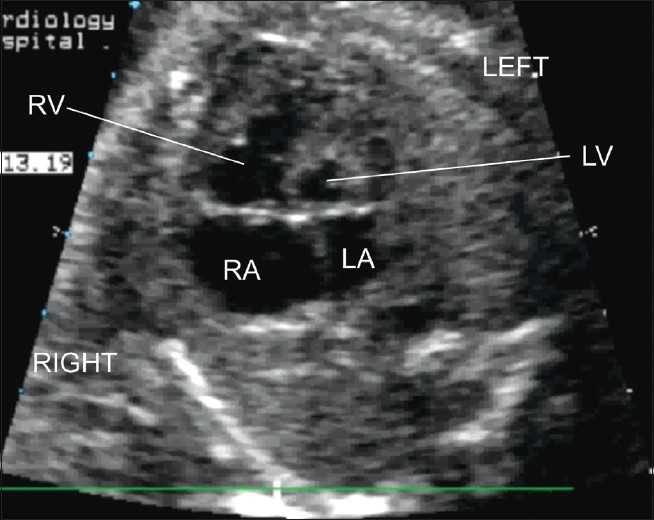
Hypoplastic left heart syndrome. The four chamber view is abnormal with a globular and hypoplastic left ventricle. The right ventricle forms the cardiac apex

**Figure 2(c):**
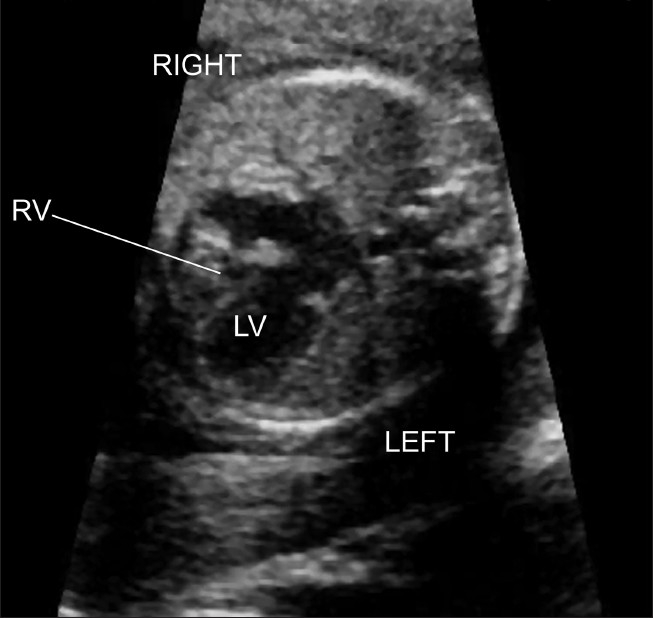
Pulmonary atresia with intact ventricular septum. The four chamber view demonstrates that the left ventricle is far larger than the right ventricle. The right ventricle is hypoplastic with a diminutive right ventricular cavity

**Figure 3(a):**
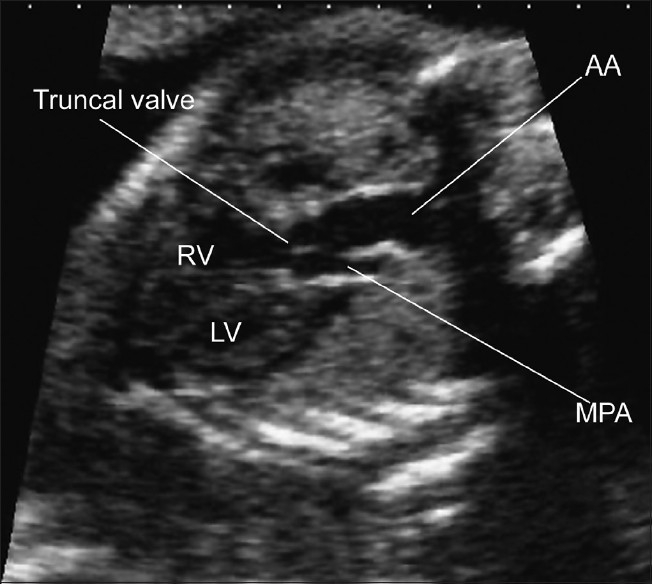
Common arterial trunk. The four chamber view was normal in this fetus. There is a single arterial trunk, which divides into the main pulmonary artery and the aorta

**Figure 3(b):**
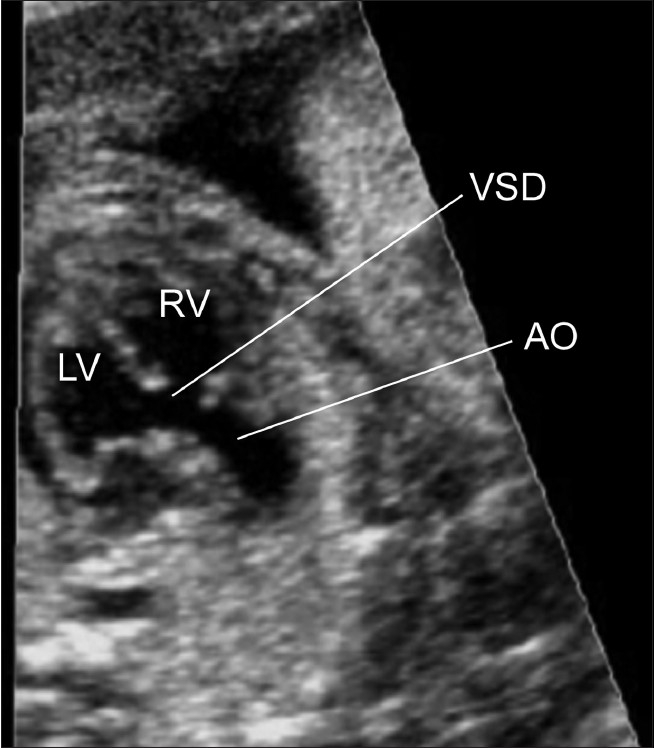
Tetralogy of Fallot. In this view the aorta can be seen to arise astride a large ventricular septal defect. The four chamber view did not demonstrate any abnormality

**Figure 3(c):**
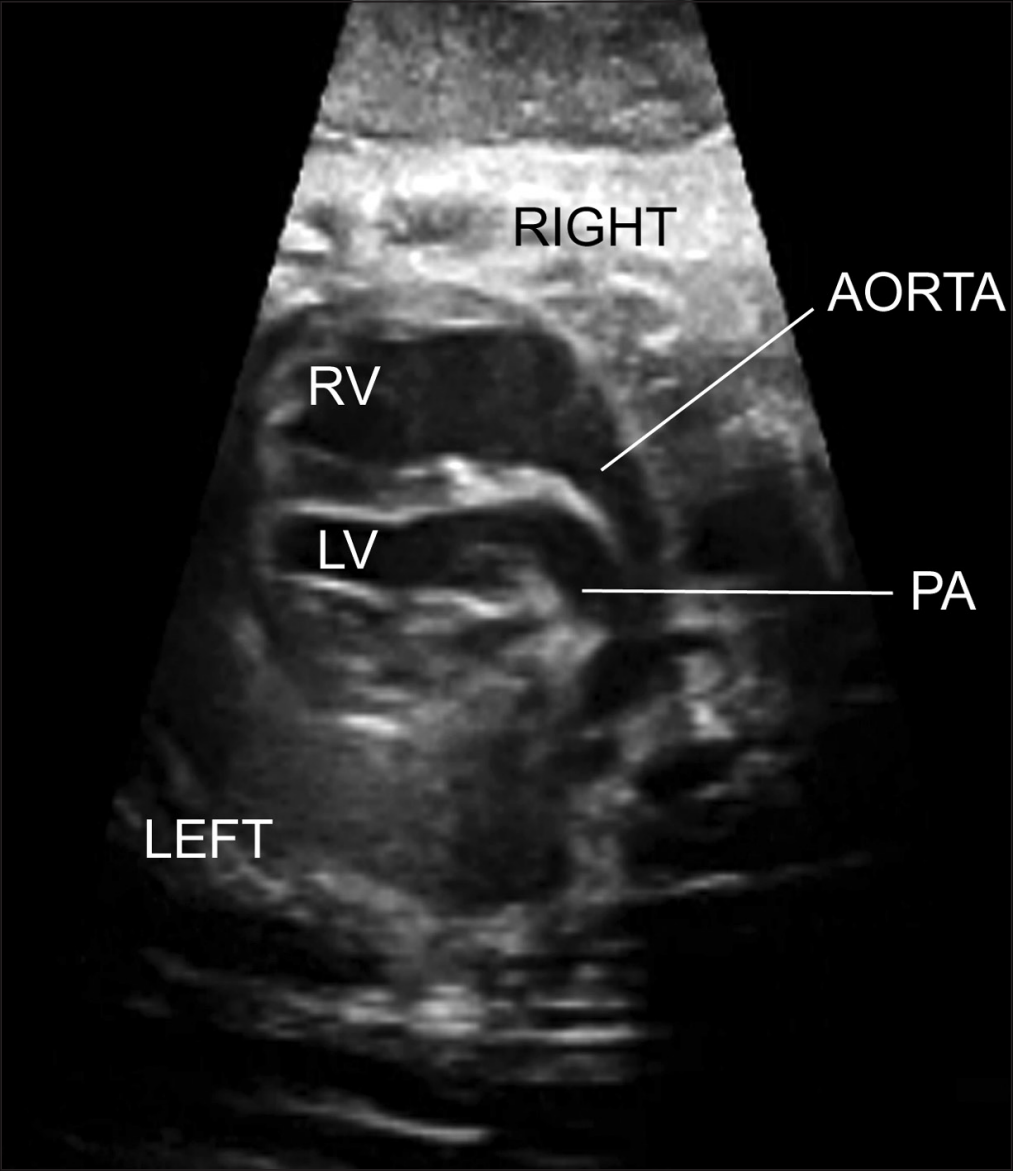
Transposition of the great arteries. The four chamber view was normal. The great arteries run parallel to the aorta, anterior to the pulmonary artery

The question of how to train sonographers to examine the fetal heart is challenging, both for the sonographer and for those who would provide such training. In terms of prenatal detection rates, the benefit of sonographer training has been described,[51–53] as well as the impact of sonographer experience[54] and variability of performance following training.[55].Whether such training is delivered by cardiologists with expertise in fetal diagnosis or obstetricians or sonographers with particular expertise will depend on local factors. It is also important to distinguish the detection rates that are reported by individual centers[56] from those that are population-based.[3,57] Individual center data will depend on the nature of the unit reporting their data, referral patterns, and the expertise within the unit. There is also publication selection bias toward better results. When population-based data is examined the results are usually much less impressive in terms of overall detection rate, with observation of vari ability of detection rates between centers and regions.[3]

One of the major limitations for using fetal echocardiography as a screening tool is that it is operator-dependent, in a way that other forms of pregnancy screening, for example, serum screening for major trisomies, are not. Published data has confirmed the influence of operator training and experience[54,55] on the ability to confirm normality of the cardiac connections. Sonographers frequently report that they find imaging of the fetal heart one of the most challenging aspects of prenatal anomaly scanning. For countries where anomaly scanning in general is not well-established, it may be most appropriate to initially incorporate some core views of the heart, such as, the four chamber view, into fetal anomaly scans before aspiring to more extensive cardiac imaging as part of anomaly scans, in the low-risk population. With time and training, operators may be more confident about the views of the outflow tracts, visualisation of which will improve overall detection rates.

## TECHNICAL DEVELOPMENTS

In recent years, technical advances in three-dimensional echocardiography show that it is possible to include all fetal cardiac structures within a “volume” of the fetal heart. The most common technique employed is spatiotemporal image correlation (STIC), which involves a slow sweep of the ultrasound probe in a pyramid that includes all cardiac structures. Such sweeps typically take 7-12 seconds to achieve a full volume, which can be obtained with or without color flow Doppler.[58] The software algorithm permits a display of multiple moving “slices” of the heart to show the anatomy from situs views below the heart, the four chamber view, and views of the outflow tracts. By incorporating the views within a single volume these can be interrogated retrospectively in any desired projection.[59,60] Although attractive, retrospective analysis does take time[61] and is dependent on the image quality in common with all ultrasound techniques. Furthermore, the relatively slow acquisition times mean that fetal movement can render some volumes useless and acoustic shadows interfere with image quality in exactly the same way as standard cross-sectional techniques. Several studies have demonstrated that such datasets can be interpreted retrospectively either to confirm normality or confirm congenital heart disease. Datasets can be sent to remote sites to be interrogated retrospectively by fetal cardiologists, which may assist screening centers that are geographically remote from the specialist centers.[62,63] Regardless of the technique that is used to obtain or analyze images of the fetal heart, an accurate interpretation of such images will depend on a firm understanding of the anatomy of the fetal heart, either to diagnose a congenital abnormality or to confirm normality. Currently, the role of STIC in “routine” screening for congenital heart defects has not been established.
